# Theoretical Calculation on the Reaction Mechanisms, Kinetics and Toxicity of Acetaminophen Degradation Initiated by Hydroxyl and Sulfate Radicals in the Aqueous Phase

**DOI:** 10.3390/toxics9100234

**Published:** 2021-09-25

**Authors:** Mengmeng Xu, Junfang Yao, Simei Sun, Suding Yan, Jingyu Sun

**Affiliations:** 1 Hubei Key Laboratory of Pollutant Analysis & Reuse Technology, College of Chemistry and Chemical Engineering, Hubei Normal University, Cihu Road 11, Huangshi 435002, China; xumm@stu.hbnu.edu.cn (M.X.); yaojf@stu.hbnu.edu.cn (J.Y.); 2 Huangshi Key Laboratory of Photoelectric Technology and Materials, College of Physics and Electronic Science, Hubei Normal University, Huangshi 435002, China; simeisun@hbnu.edu.cn; 3 College of Urban and Environmental Sciences, Hubei Normal University, Huangshi 435002, China; yansd@hbnu.edu.cn

**Keywords:** acetaminophen (AAP), density functional theory, degradation mechanisms, rate constants, acute toxicity, chronic toxicity

## Abstract

The •OH and SO_4_^•−^ play a vital role on degrading pharmaceutical contaminants in water. In this paper, theoretical calculations have been used to discuss the degradation mechanisms, kinetics and ecotoxicity of acetaminophen (AAP) initiated by •OH and SO_4_^•−^. Two significant reaction mechanisms of radical adduct formation (RAF) and formal hydrogen atom transfer (FHAT) were investigated deeply. The results showed that the RAF takes precedence over FHAT in both •OH and SO_4_^•−^ with AAP reactions. The whole and branched rate constants were calculated in a suitable temperature range of 198–338 K and 1 atm by using the KiSThelP program. At 298 K and 1 atm, the total rate constants of •OH and SO_4_^•−^ with AAP were 3.23 × 10^9^ M^−1^ s^−1^ and 4.60 × 10^10^ M^−1^ s^−1^, respectively, considering the diffusion-limited effect. The chronic toxicity showed that the main degradation intermediates were harmless to three aquatic organism, namely, fish, daphnia, and green algae. From point of view of the acute toxicity, some degradation intermediates were still at harmful or toxic level. These results provide theoretical guidance on the practical degradation of AAP in the water.

## 1. Introduction

The problem of water pollution caused by the drug residues have been paid much more attention. Even though the content of these drugs in the water environment is very low, they brings potential dangers to human health and ecological environment due to its strong persistence, bioaccumulation and slow biodegradation [1,2]. Acetaminophen (AAP), as one kind of antipyretic analgesics, enters to water environment by the excretion of humans and animals. The concentration of AAP rose to 6 μg/L in European STP effluents [3]. Its concentration up to 10 μg/L was detected in natural waters in the United States [4], and over 65 μg/L concentration was measured in the Tyne river in the United Kingdom [5]. The removal of micropollution is challenging for water treatment technology. Researches on this area were also relatively limited.

Advanced oxidation processes (AOPs) are highly efficient engineering technologies in the elimination of water micropollutants. The active free radicals (i.e., •OH and SO_4_^•−^) generating in AOPs can decompose these contaminants. The ultraviolet/hydrogen peroxide (UV/H_2_O_2_), Fenton (H_2_O_2_/Fe^2+^) and Photo-Fenton (UV/H_2_O_2_/Fe^2+^) processes can contribute to •OH, which is shown in the following equation:

Fe^2+^ + H_2_O_2_ → Fe^3+^ + •OH + HO^−^

•OH could be generated from hydrogen peroxide activated by electrochemical process with Fe^2+^/Fe^3+^, which can degrade pentachlorophenol (PCP) [6]. The removal of carbamazepine (CBZ) was attributed to •OH formed by coupling H_2_O_2_ with UV and Fe^2+^/Fe^3+^ [7]. The removal efficiency of three AOP systems has been compared and found the order of O_3_/H_2_O_2_/Fe^2+^ > UV/H_2_O_2_/Fe^2+^ > H_2_O_2_/Fe^2+^ [8]. Certainly, other processes such as photocatalysis and photoelectrocatalysis are able to produce •OH [9].

Meanwhile, the ultraviolet/persulfate (UV/S_2_O_8_^2−^) can generate SO_4_^•−^, which is described by the following equation [10]:

$$S_{2}O_{8}^{2-}\overset{hv}{\to }{\;\mathrm{SO}}_{4}^{•-}+{\;\mathrm{SO}}_{4}^{•-}$$

Surely, •OH can be produced when SO_4_^•−^ reacts with H_2_O, which is presented by the following equation [11]:

$${\mathrm{SO}}_{4}^{•-}+{\;H}_{2}O\;\to {\;\mathrm{SO}}_{4}^{2-}+•\mathrm{OH}\;+{\;H}^{+}$$

Thus, •OH-mediated and SO_4_^•−^-mediated degradation of contaminants were available. The redox potential and rate constants are summarized in Table 1. It was reported that the •OH-initiated degradation rate constant was about 10^8^–10^10^ M^−1^ s^−1^. The SO_4_^•−^-initiated rate constant was about 10^7^–10^10^ M^−1^ s^−1^ [12]. The degradation rates of two reactive radicals are nearly equivalent, which is consistent with their high redox potential (2.5–3.1 V for SO_4_^•−^ versus 1.8–2.7 V for •OH) [13,14,15]. The degradation processes of contaminants triggered by •OH and SO_4_^•−^ were investigated in recent years. For example, Tong et al. determined the rate constants of syringic acid reactions with •OH and SO_4_^•−^ in aqueous phase by laser flash photolysis. They found that •OH and SO_4_^•−^ possessed similar reaction rate at the same pH [16]. Gao et al. measured the rate constants of neutral sulfamethoxazole with •OH and SO_4_^•−^ were (7.27 ± 0.43) × 10^9^ and (2.98 ± 0.32) × 10^9^ M^−1^ s^−1^ in the systems of UV/H_2_O_2_ and UV/PS, respectively [17]. Similarly, Wang et al. detected the rate constants for AAP with •OH and SO_4_^•−^ reactions were (3.26 ± 0.41) × 10^9^ and (1.80 ± 0.17) × 10^9^ M^−1^ s^−1^ in the Fe^2+^/persulfate system, respectively [18]. The second-order rate constants of •OH and SO_4_^•−^ were conformed as 5.15 × 10^9^ and 7.66 × 10^9^ M^−1^ s^−1^, respectively, using the ultraviolet light emitting diode (UV-LED)-based method by Li et al. [19]. However, the study of degradation mechanisms of •OH and SO_4_^•−^ with the target contaminants still faced with great challenge. At atom level, quantum chemistry calculation was a powerful tool to gain a in-depth understanding for mechanisms and kinetics of •OH and SO_4_^•−^ reacting with some pollutants [20,21,22,23]. 

Theoretical studies are essential for discussing the degradation processes of AAP with •OH and SO_4_^•−^. Therefore, the reaction mechanisms and kinetics of the AAP with •OH and SO_4_^•−^ have been studied by using quantum chemistry calculations. Rate constants of every possible pathways for AAP with •OH and SO_4_^•−^ reactions were calculated. Even more importantly, the ecotoxicity of AAP and its degradation products has been evaluated in order to know their risk.

## 2. Computational Methods

### 2.1. Mechanism Calculation

Usually, reaction mechanisms are investigated by Density functional theory (DFT). M06-2X functional can solve noncovalent interactions for some complexes better than other density functional such as B3LYP [24]. The functional ratio of exchange correction of M06-2X is 54% which will obtain more accurate energies [25]. Furthermore, M06-2X method [26] of DFT was selected in the reactions of AAP with •OH and SO_4_^•−^ without hesitation, because satisfactory results were acquired on the degradation of some micropollutants [27,28,29,30]. For example, the thermodynamic and kinetic data for ibuprofen reactions with hydroxyl and sulfate radicals reported by Yang et al. were calculated with M06-2X method [27]. All electronic structures and energy calculations were performed using Gaussian 09 software [31]. The reactants (R), transition states (TS) and intermediates (IM) were optimized at the M06-2X/6-31+G(d,p) level. IM (all positive frequencies) and TS (only one imaginary frequency) are primarily identified by harmonic vibration frequency analysis. Moreover, the method of intrinsic reaction coordinates (IRC) was applied to determine every right transition state [32]. The water solvent effect was taken into account by a universal solvation model (SMD) [33] when these structures were optimized in the aqueous phase. Based on right structures, the single point energies were calculated accurately at high level of M06-2X/6-311++G(3df, 2p).The Gibbs free energy barrier of activation (Δ*G^≠^*) and free energy of reaction (Δ*G*) are calculated as follows:

Δ*G^≠^* = *G*_TS_ − *G*_R_

Δ*G* = *G*_IM_ − *G*_R_

### 2.2. Kinetic Calculation

The conventional Transition State Theory (TST) was used to calculate the rate constants implemented by KiSThelP program [34] that has obtained accurate results for contaminants with free radicals reactions [35,36,37,38,39]. The calculation formula is employed in KiSThelP:

$$k=\kappa \sigma \frac{k_{b}T}{h}{\left(\frac{RT}{P^{0}}\right)}^{\Delta n}e^{-\frac{\Delta G^{0,\neq }}{k_{b}T}}$$

Some parameters need to be explained. κ is tunneling correction factor of Wigner approach [34]. σ, *k_b_* and *h* are the reaction path degeneracy, Boltzmann’s constant and Planck’s constant, respectively. ∆*G*^0,≠^ is the standard Gibbs free energy of activation. RT/*P*^0^ has the unit of the inverse of a concentration. For bimolecular reactions, ∆*n* is equal to 1.

The diffusion-limited effect was considered to obtain the apparent rate constants (*k*_app_) of aqueous phase based on Collins-Kimball theory [40].

$$k_{app}=\frac{k_{aq}k_{D}}{k_{aq}+k_{D}}$$

where, *k_aq_* is calculated by TST as aqueous rate constant. *k_D_* is calculated by the Smoluchowski equation as the diffusion-limited rate constants:

$$k_{D}=4\pi R_{AB}D_{AB}N_{A}$$

*R_AB_* means the reaction distance, and *N*_A_ is Avogadro’s number, *D_AB_* represents the sum of diffusion coefficient of the reactants A (AAP) and B (•OH or SO_4_^•−^). The calculations of *D_A_* and *D_B_* are realized by using the Stokes–Einstein approach [41]:

$$D=\frac{k_{b}T}{6\pi \alpha \eta }$$

where *k*_b_, *T*, *η*, and *α* are the Boltzmann constant, temperature, viscosity of the solvent, and radius of the solute, respectively. For water, *η* = 8.9 × 10^−4^ Pa s.

### 2.3. Ecotoxicity Calculation

The aquatic toxicity of AAP and its degradation products was evaluated by using the Structure Activity Relationship (SAR) method with the ECOSAR program [42], which has been successfully used to evaluate the acute and chronic toxicity [43,44,45,46]. Three aquatic organisms of green algae, daphnia and fish were considered to assess the acute and chronic toxicities. Acute toxicity of the target compounds was estimated by median lethal concentration (LC50) and median effect concentration (EC50). LC50 is defined 50% lethal concentration for fish and daphnia in 96 and 48 h, respectively. EC50 is 50% effective concentration for green algae in 96 h. The chronic toxicity was described by the chronic toxicity value (ChV) for green algae, daphnia and fish.

## 3. Results and Discussion

### 3.1. Degradation Mechanisms

**Figure 1:**
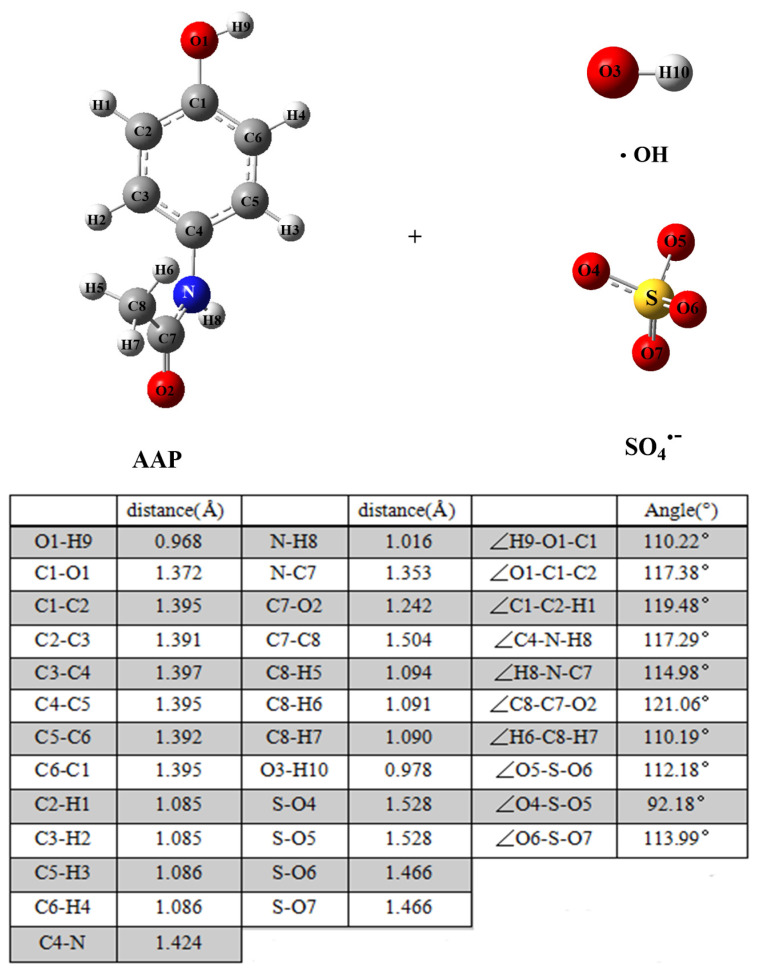
The structures of AAP, •OH and SO_4_^•−^ calculated at the M06-2X/6-31 + G(d,p) level. The bond distance (Å) and angles (°) are listed. Here, 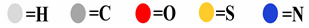.

The degradation mechanisms of AAP initiated by •OH and SO_4_^•−^ mainly include radical adduct formation (RAF) and formal hydrogen atom transfer (FHAT). Similarities and differences of mechanisms about two reactions were adequately investigated. The Gibbs free energy of reaction (Δ*G*) and Gibbs free energy barrier of activation (Δ*G^≠^*) of the initial reaction of AAP with •OH and SO_4_^•−^ were calculated and discussed. The binding distances and angles of AAP, •OH and SO_4_^•−^ are shown in Figure 1. All structures of transition states are plotted in Figure S1 (Supplementary Materials).

#### 3.1.1. Radical Adduct Formation

**Figure 2:**
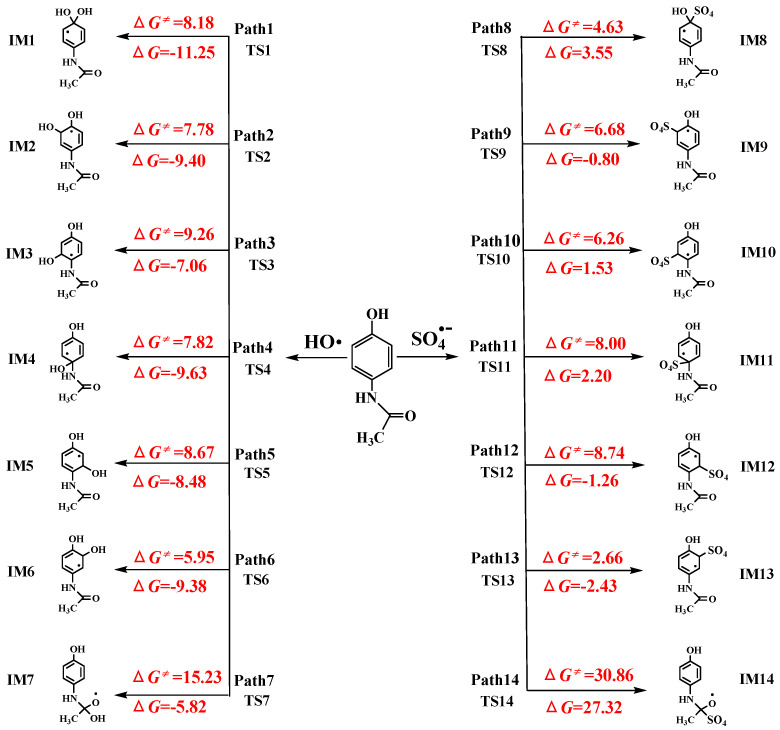
The radical adduct formation channels of AAP with •OH and SO_4_^•−^ reactions with the Gibbs free energy of reaction (Δ*G*) and Gibbs free energy barrier of activation (Δ*G^≠^*) (unit: kcal/mol).

**Figure 3:**
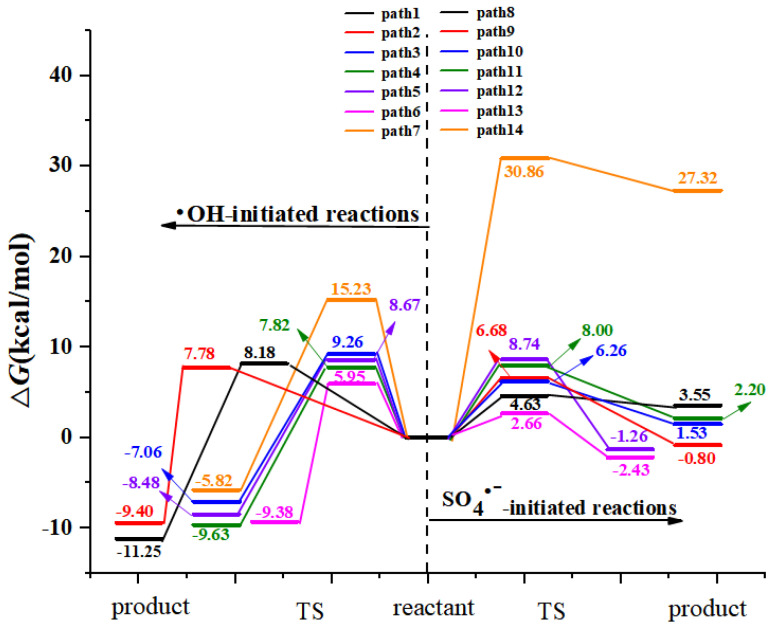
The free energy diagram of RAF pathways initiated by two radicals.

RAF pathways of AAP with •OH and SO_4_^•−^ reactions are displayed in Figure 2. •OH-initiated and SO_4_^•−^-initiated RAF channels consist of addition on the benzene ring and the acetamide group. It is uniform for the RAF mechanisms of AAP with •OH and SO_4_^•−^ reactions. The acetamide group addition has no advantage over that of the benzene ring because the free energy barriers are 15.23 and 30.86 kcal/mol for acetamide group addition of •OH and SO_4_^•−^, respectively. However, the free energy barriers were 5.95–9.26 kcal/mol and 2.66–8.74 kcal/mol for •OH and SO_4_^•−^ addition to six carbon atoms of benzene ring, respectively. Generally, •OH-triggered reactions are higher exothermic than that of SO_4_^•−^. Based on the values of Δ*G^≠^* and Δ*G*, C_6_ atom addition (path 6 for •OH-triggered reactions versus path 13 for SO_4_^•−^-triggered reactions) is the most favorable channels because their barriers are only 5.95 and 2.66 kcal/mol, respectively. Recently, the similar addition results were proved by Li et al. [47]. Figure 3 shows the comparison of potential energies for RAF mechanisms of two radicals reactions. SO_4_^•−^-initiated reactions have the lower free energy barriers than that of •OH. In SO_4_^•−^-initiated reactions, TS13, C_6_ addition transition state, has stronger hydrogen bond intermolecular interaction, namely, hydrogen atom of phenolic hydroxyl group of AAP with oxygen atom of SO_4_^•−^. IRC intuitively shows hydrogen atom of phenolic hydroxyl group of AAP tends to be close to oxygen atom of SO_4_^•−^. The interaction will greatly decrease reaction barrier. However, SO_4_^•−^-initiated reactions have less reaction heats compared with •OH-initiated reactions. For example, the energy barrier of path 6 is higher 3.29 kcal/mol than path 13, but path 6 is more exothermic than 6.95 kcal/mol.

#### 3.1.2. Formal Hydrogen Atom Transfer

**Figure 4:**
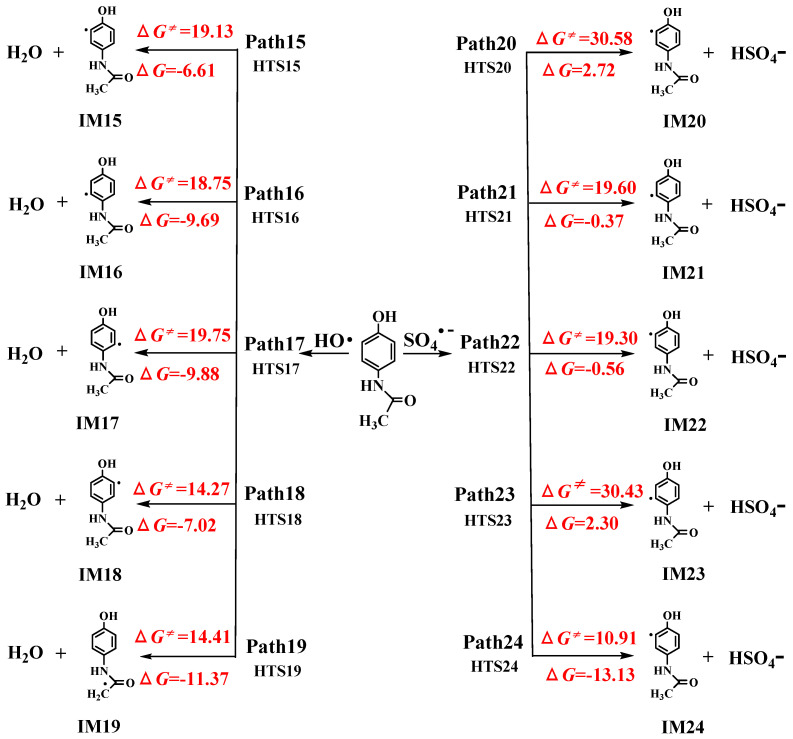
The formal hydrogen atom transfer channels of AAP with •OH and SO_4_^•−^ reactions with the Gibbs free energy of reaction (Δ*G*) and Gibbs free energy barrier of activation (Δ*G^≠^*) (unit: kcal/mol).

**Figure 5:**
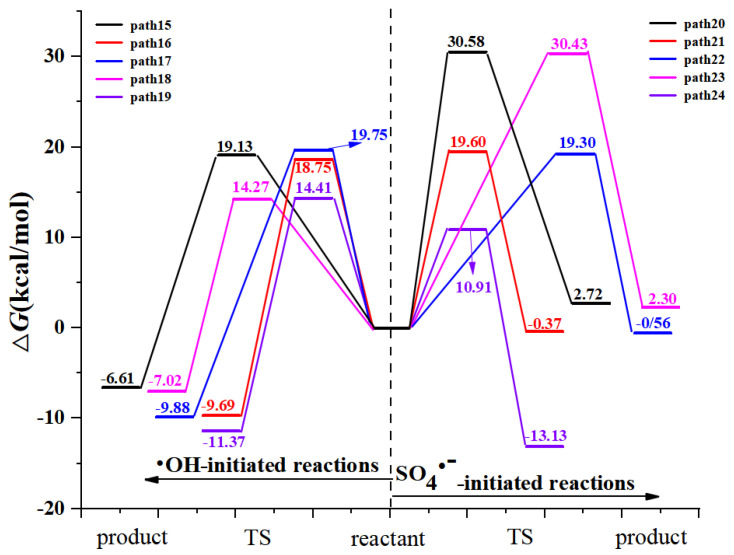
The free energy diagram of FHAT pathways initiated by two radicals in the aqueous phase.

Ten hydrogen abstraction pathways from benzene ring and methyl group are found and shown in Figure 4. Hydrogen abstractions from C2, C3, and C5 of benzene ring experience TS15, TS16, TS17 with free energy barriers of 19.13, 18.75 and 19.75 kcal/mol for AAP with •OH reactions, respectively. For SO_4_^•−^-initiated reactions, the free energy barriers of hydrogen abstractions from C2, C3, C5, and C6 of benzene ring are 30.58, 19.60, 19.30 and 30.43 kcal/mol, respectively. The hydrogen atom can be abstracted from C_6_ of benzene ring and the methyl group via 14.27 and 14.41 kcal/mol barriers for •OH-initiated path 18 and path 19. Moreover, the corresponding products are exothermic 7.02 and 11.37 kcal/mol, respectively. The results indicate that hydrogen abstractions from C_6_ of benzene ring and methyl group are two important channels for •OH with AAP reaction. However, methyl group hydrogen abstraction is the most important channel for SO_4_^•−^ with AAP reaction due to the lowest energy barrier of 10.91 kcal/mol. Figure 5 describes the comparison of free energies for FHAT mechanisms. As shown in the Figure 5, the free energy barriers for SO_4_^•−^ abstracting hydrogen atom from benzene ring are higher than that of •OH, and the corresponding paths (path 20–path 23) initiated by SO_4_^•−^ are less exothermic than path 15–path 18 initiated by •OH. However, the free energy barrier of SO_4_^•−^-initiated path 24 is lower 3.5 kcal/mol than •OH-initiated path 19, and path 24 is more exothermic 1.76 kcal/mol than path 19.

**Figure 6:**
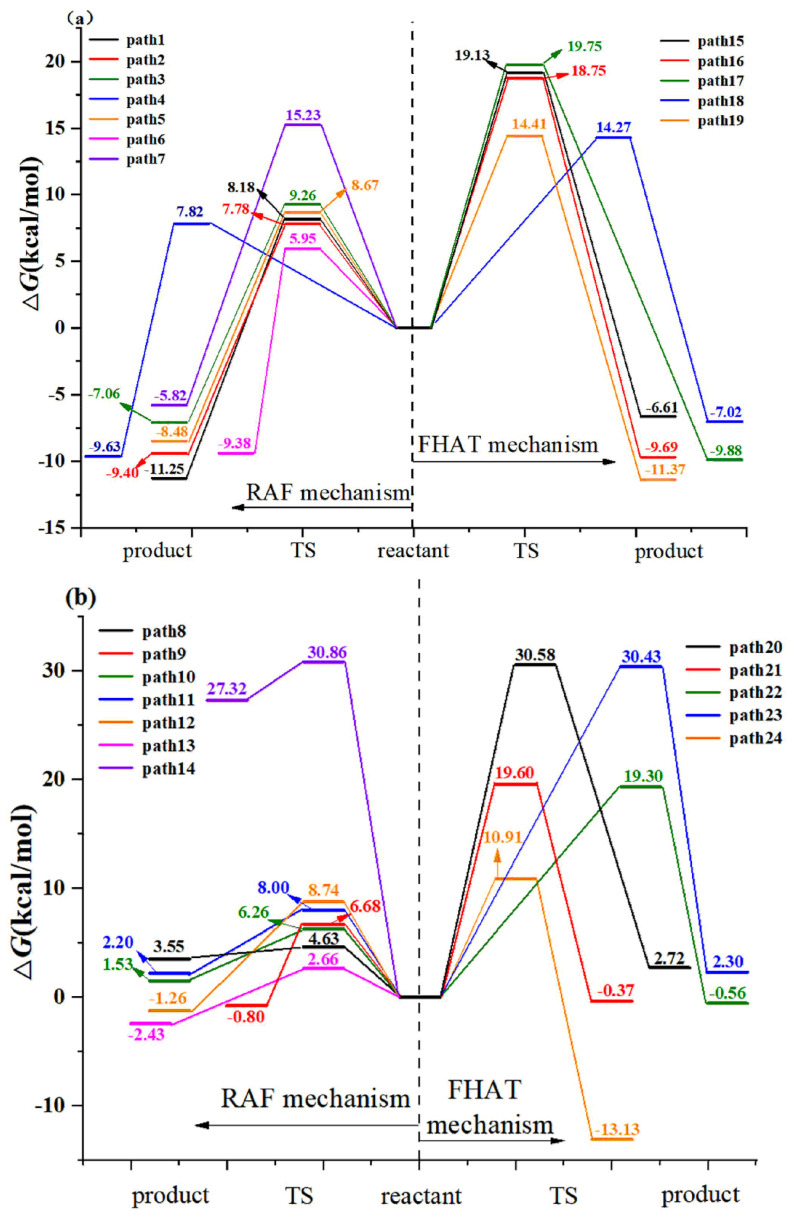
Free energy profiles for FHAT and RAF mechanisms in aqueous phase. (**a**) APP with •OH reactions; (**b**) APP with SO_4_^•−^ reactions.

The comparison of FHAT and RAF mechanisms is shown in Figure 6. It is implied that RAF has an advantage over FHAT for both reactions. The free energy barrier for the most important RAF channel is lower 8.32 and 8.25 kcal/mol than the most favorable FHAT channel for •OH-initiated and SO_4_^•−^-initiated reactions, respectively.

### 3.2. Kinetics

The rate constants involved free radicals reactions are of great value for predicting the degradation rate. However, the measurement of such data is difficult due to these rapid reactions. The theoretical calculations play an important role in attaining rate constants for these radical-participating reactions.

The rate constants for AAP with •OH reactions are given in Table 2. The apparent rate constant of •OH reaction with AAP (*k*_app_) is 3.23 × 10^9^ M^–1^ s^–1^ at 298 K. The calculated rate constant is consistent with experimental results of (3.26 ± 0.41) × 10^9^ and 5.15 × 10^9^ M^–1^ s^–1^ [18,19]. The C_6_ site addition (path 6) has the largest rate constant of 3.56 × 10^9^ M^–1^ s^–1^ with the 84.8% branching ratio. The other RAF and FHAT pathways contribute the total reactions weakly. The rate constants for AAP with SO_4_^•−^ reactions are depicted in Table 3. The apparent rate constant of SO_4_^•−^ reaction with AAP (*k’*_app_) is 4.60 × 10^10^ M^–1^ s^–1^ at 298 K, which is higher six times than experimental value of 7.66 × 10^9^ M^–1^ s^–1^ [19]. The possible reason is that the lower barrier leads to higher reaction rate, which agrees with discussion of mechanisms. Theoretical model and method will lead to some deviations, but the accuracy of experiment is affected by some factors such as equipment, reagent, and operation. Theoretical calculations can predict and explain some results. Consequently, the benefits of theoretical calculations cannot be underestimated. The C_6_ site addition (path 13) is dominant channel with the largest rate constant of 8.65 × 10^13^ M^–1^ s^–1^ that possesses the 92.8% branching ratio. The other RAF and FHAT pathways have a little contribution for AAP with SO_4_^•−^ reaction. As shown in Table 4, C_6_ of benzene ring and methyl group hydrogen abstractions are dominant channels for •OH with AAP reaction with the branching ratio of 50.42% and 49.58%, respectively. For AAP with SO_4_^•−^ reaction, methyl group hydrogen abstraction contributes 100% to FHAT channels.

**Figure 7:**
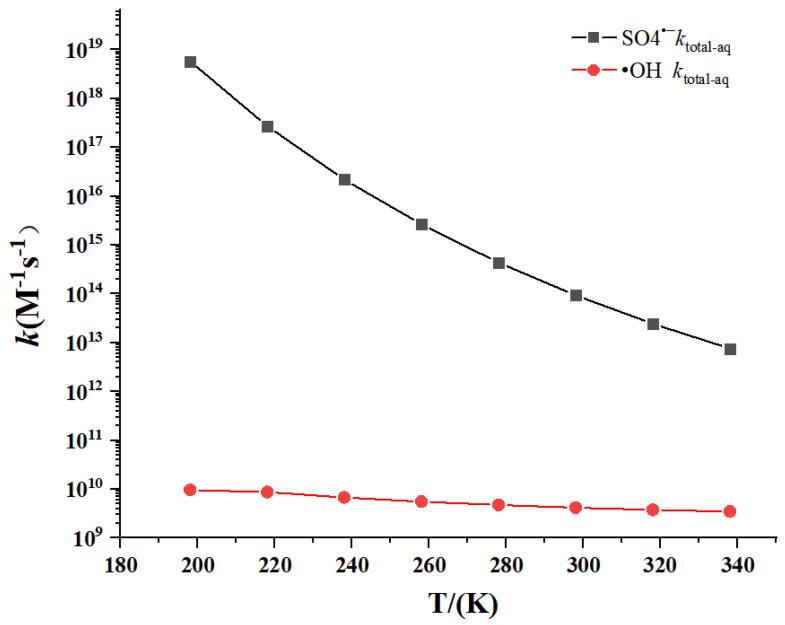
Temperature dependence of the calculated rate constants.

The temperature dependence of rate constants is shown in Figure 7 at the temperatures from 198 to 338 K and 1 atm, and the corresponding data are listed in Tables S1 and S2 (Supplementary Materials). The total rate constants have weakly negative temperature dependence for •OH-initiated reaction. However, SO_4_^•−^-initiated reactions have distinctly negative temperature dependence.

### 3.3. The Aquatic Toxicities of AAP and Its Degradation Intermediates

**Figure 8:**
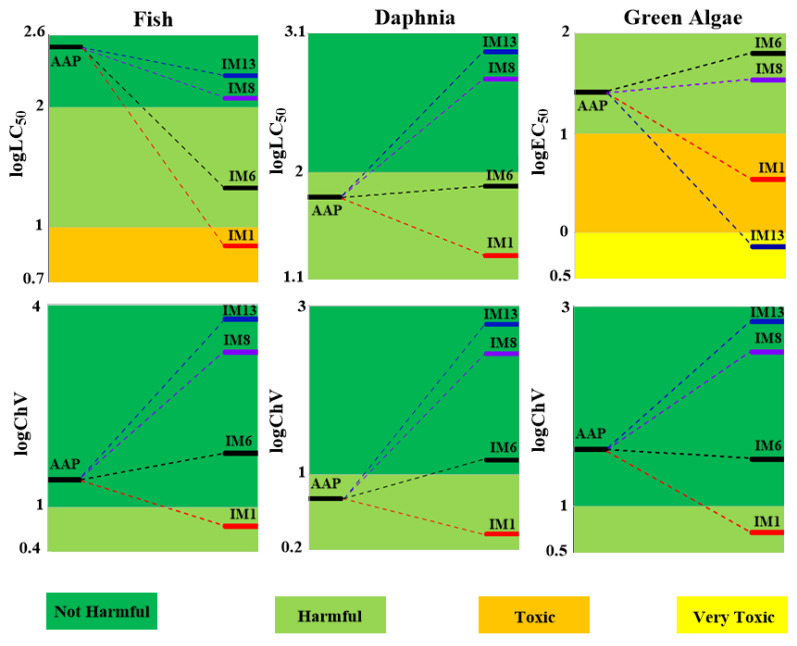
Acute and chronic toxicity (mg/L) of AAP and its transformation intermediates to aquatic organisms (fish, daphnia, and green algae).

The acute and chronic toxicities of AAP and the important degradation intermediates are assessed in three different aquatic organisms, which is drawn in Figure 8. Four types are classified and listed in Table S3 (Supplementary Materials). The toxic values of AAP and the important degradation intermediates are shown in Table S4 (Supplementary Materials).

#### 3.3.1. Toxicity of AAP

The acute toxicity value of AAP is calculated as 323 mg/L of LC50 for fish, 63.1 mg/L of LC50 for daphnia and 26.3 mg/L of EC50 for green algae, respectively. These results indicate that AAP is harmful to daphnia and green algae, but not harmful to fish. The calculated ChV of AAP is 26.3 mg/L for fish, 5.13 mg/L for daphnia, and 37.2 mg/L for green algae. AAP is not harmful to fish and green algae at chronic level. However, it is harmful to daphnia chronically.

#### 3.3.2. Toxicities of the Degradation Products

The most important intermediate (IM6) is harmful to three aquatic organisms in acute toxicity, but is harmless to three aquatic organisms in chronic toxicity. For other degradation intermediates, IM1 is acutely toxic for fish and green algae, and harmful to daphnia. The chronic toxicity of IM1 is harmful for three aquatic organisms. IM13 and IM8 are not harmful for three aquatic organisms chronically. Moreover, IM13 and IM8 are not acutely harmful for fish and daphnia, but pose a severe threat for green algae. In brief, the most important degradation intermediate (IM6) from •OH-initiated reaction is still harmful to aquatic organisms. IM13 from SO_4_^•−^-initiated reaction is harmless to fish and daphnia, but is very toxic to green algae. Thus, the toxicity of these compounds should be concerned.

## 4. Conclusions

In this work, the reaction mechanisms and rate of AAP with •OH and SO_4_^•−^ have been explored theoretically in aqueous phase. The toxicity of AAP and its transformation intermediates to three aquatic organisms have been assessed. The novelty are summarized as below:

(1) M06-2X/6-311+G (3df, 2p)//M06-2X/6-31+G (d, p) has been used to study the •OH-initiated and SO_4_^•−^-initiated transformation mechanism of AAP. •OH and SO_4_^•−^ with AAP reactions have the same reaction sites, even reaction mechanisms. The results implied that the C_6_ addition is prominent pathway in RAF mechanisms and hydrogen abstraction of methyl group is dominant pathway for both reactions in FHAT mechanism. RAF takes precedence over FHAT.
(2) At 298 K, the total apparent rate constant of AAP with SO_4_^•−^ is larger than that of •OH. The calculated rate constants basically matched with experimental values. Theoretical calculations predicted the kinetic data at 198 K–338 K.
(3) Toxic assessment shows that some representative degradation intermediates present an acute threat to the target organisms. Thus, subsequent degradation should be implemented until they are degraded into non-toxic substances.

In brief, this work explains the degradation processes of AAP initiated by •OH and SO_4_^•−^ from microscopic points, and solves the problem of structures of intermediates and products which are associated with reactivity. The calculation of eco-toxicity plays an important role on assessing toxicity of degradation process. Finally, these results can apply to the practical degradation of AAP in AOPs.

## Figures and Tables

**Table 1:** Redox potential and kinetic data for the reactions of •OH and SO_4_^•−^.

Radicals	Redox Potential ^a^ (V)	The Range of Rate Constants ^b^ (M^−1^ s^−1^)	The Second-Order Rate Constants of Neutral Sulfamethoxazole ^c^ (M^−1^ s^−1^)	The Second-Order Rate Constants of Acetaminophen (M^−1^ s^−1^)	
				Fe^2+^/PS ^d^	UV-LED/H_2_O_2_ ^e^
•OH	1.8–2.7	10^8^–10^10^	(7.27 ± 0.43) × 10^9^	(3.26 ± 0.41) × 10^9^	5.15 × 10^9^
SO_4_^•−^	2.5–3.1	10^7^–10^10^	(2.98 ± 0.32) × 10^9^	(1.80 ± 0.17) × 10^9^	7.66 × 10^9^

^a^ (Xiao, et al., 2020; Devi, et al., 2016; Ghanbari, et al., 2017); ^b^ (Li, et al. 2020, [12]); ^c^ (Gao, et al., 2020); ^d^ (Wang, et al., 2019); ^e^ (Li, et al., 2020, [19]).

**Table 2:** The calculated rate constants(*k*_aq_), steady-state rate constant (*k*_D_), apparent rate constant (*k*_app_) and branching ratio (*R*_aq_) for the AAP with •OH reaction in the aqueous phase at 298 K.

Paths	*k*_aq_ (M^−1^ s^−1^)	*R*_aq_ (%)	*k*_D_ (M^−1^ s^−1^)	*k*_app_ (M^−1^ s^−1^)
APP + •OH → IM1 (*k*_1_)	8.04 × 10^7^	1.9	9.80 × 10^9^	7.97 × 10^7^
APP + •OH → IM2 (*k*_2_)	1.87 × 10^8^	4.5	9.80 × 10^9^	1.83 × 10^8^
APP + •OH → IM3 (*k*_3_)	1.51 × 10^7^	0.4	9.80 × 10^9^	1.51× 10^7^
APP + •OH → IM4 (*k*_4_)	3.22 × 10^8^	7.6	9.80 × 10^9^	3.12 × 10^8^
APP + •OH → IM5 (*k*_5_)	3.33 × 10^7^	0.8	9.80 × 10^9^	3.32 × 10^7^
APP + •OH → IM6 (*k*_6_)	3.56 × 10^9^	84.8	9.80 × 10^9^	2.61 × 10^9^
APP + •OH → IM7 (*k*_7_)	6.75 × 10^2^	0	9.80 × 10^9^	6.75 × 10^2^
APP + •OH → IM15 (*k*_15_)	4.75	0	9.80 × 10^9^	4.75
APP + •OH → IM16 (*k*_16_)	9.75	0	9.80 × 10^9^	9.75
APP + •OH → IM17 (*k*_17_)	1.91	0	9.80 × 10^9^	1.91
APP + •OH → IM18 (*k*_18_)	1.15 × 10^4^	0	9.80 × 10^9^	1.15 × 10^4^
APP + •OH → IM19 (*k*_19_)	1.13 × 10^4^	0	9.80 × 10^9^	1.13 × 10^4^
APP + •OH → Product (*k*_total_)	4.20 × 10^9^	100		3.23 × 10^9^

**Table 3:** The calculated rate constants(*k’*_aq_), steady-state rate constant (*k’*_D_), apparent rate constant (*k’*_app_) and branching ratio (*R’*_aq_) for the AAP with SO_4_^•−^ reaction in the aqueous phase at 298 K.

Paths	*k’*_aq_ (M^−1^ s^−1^)	*R’*_aq_ (%)	*k’*_D_ (M^−1^ s^−1^)	*k’*_app_ (M^−1^ s^−1^)
APP + SO_4_^•−^→IM8 (*k’*_8_)	6.00 × 10^12^	6.4	8.05 × 10^9^	8.04 × 10^9^
APP + SO_4_^•−^ → IM9 (*k’*_9_)	1.61 × 10^11^	0.2	8.05 × 10^9^	7.67 × 10^9^
APP + SO_4_^•−^ → IM10 (*k’*_10_)	2.60 × 10^11^	0.3	8.05 × 10^9^	7.81 × 10^9^
APP + SO_4_^•−^ → IM11 (*k’*_11_)	3.28 × 10^10^	0.01	8.05 × 10^9^	6.46 × 10^9^
APP + SO_4_^•−^ → IM12 (*k’*_12_)	2.52 × 10^11^	0.3	8.05 × 10^9^	7.80 × 10^9^
APP + SO_4_^•−^ → IM13 (*k’*_13_)	8.65 × 10^13^	92.8	8.05 × 10^9^	8.05 × 10^9^
APP + SO_4_^•−^ → IM14 (*k’*_14_)	1.77 × 10^−6^	0	8.05 × 10^9^	1.77 × 10^−6^
APP + SO_4_^•−^ → IM20 (*k’*_20_)	14.3	0	8.05 × 10^9^	14.3
APP + SO_4_^•−^ → IM21 (*k’*_21_)	1.11 × 10^2^	0	8.05 × 10^9^	1.11 × 10^2^
APP + SO_4_^•−^ → IM22 (*k’*_22_)	1.88 × 10^2^	0	8.05 × 10^9^	1.88 × 10^2^
APP + SO_4_^•−^ → IM23 (*k’*_23_)	5.55	0	8.05 × 10^9^	5.55
APP + SO_4_^•−^ → IM24 (*k’*_24_)	1.33 × 10^8^	0	8.05 × 10^9^	1.33 × 10^8^
APP + SO_4_^•−^ → Product (*k’*_total_)	9.32 × 10^13^	100		4.60 × 10^10^

**Table 4:** The calculated rate constants (*k*_aq_, *k*’_aq_) and branching ratio (*R*_aq_, *R*’_aq_) for the formal hydrogen atom transfer channels in the aqueous phase at 298 K.

Paths	*k*_aq_ (M^−1^ s^−1^)	*R*_aq_ (%)	Paths	*k’*_aq_ (M^−1^ s^−1^)	*R’*_aq_ (%)
APP + •OH (FHAT)	2.28 × 10^4^	100	APP + SO_4_^•−^ (FHAT)	1.33 × 10^8^	100
APP + •OH → IM15 (*k*_15_)	4.75	0	APP + SO_4_^•−^ → IM20 (*k’*_20_)	14.3	0
APP + •OH → IM16 (*k*_16_)	9.75	0	APP + SO_4_^•−^ → IM21 (*k’*_21_)	1.11 × 10^2^	0
APP + •OH → IM17 (*k*_17_)	1.91	0	APP + SO_4_^•−^ → IM22 (*k’*_22_)	1.88 × 10^2^	0
APP + •OH → IM18 (*k*_18_)	1.15 × 10^4^	50.42	APP + SO_4_^•−^ → IM23 (*k’*_23_)	5.55	0
APP + •OH → IM19 (*k*_19_)	1.13 × 10^4^	49.58	APP + SO_4_^•−^ → IM24 (*k’*_24_)	1.33 × 10^8^	100

## Supplementary Materials

The following are available online at https://www.mdpi.com/article/10.3390/toxics9100234/s1, Figure S1: Optimized geometries involving the transition states of AAP with •OH and SO4^•−^ at the M06-2X/6-31+G(d,p) level, Table S1: Calculated rate constants of AAP with •OH from 198 to 338 K and 1 atm, Table S2: Calculated rate constants of AAP with SO4^•−^ from 198 to 338 K and 1 atm, Table S3: The acute and chronic toxicity class (mg L^−1^), Table S4: Eco-toxicity values of AAP and its transformation intermediates to aquatic organisms (mg L^−1^)
