# Supplementary material for: Theoretical Calculation on the Reaction Mechanisms, Kinetics and Toxicity of Acetaminophen Degradation Initiated by Hydroxyl and Sulfate Radicals in the Aqueous Phase
Source: Toxics. 2021 Sep 25;9(10):234. doi: 10.3390/toxics9100234 (PMC8537891; doi:10.3390/toxics9100234)
Supplement: Supplementary file 1 [file toxics-09-00234-s001.zip › toxics-1366120-supplementary.pdf]

toxics

MDPI

Supplementary Materials: Theoretical Calculation on the Reaction Mechanisms, Kinetics and Toxicity of Acetaminophen Degradation Initiated by Hydroxyl and Sulfate Radicals in the Aqueous Phase

Mengmeng Xu, Junfang Yao, Simei Sun, Suding Yan and Jingyu Sun

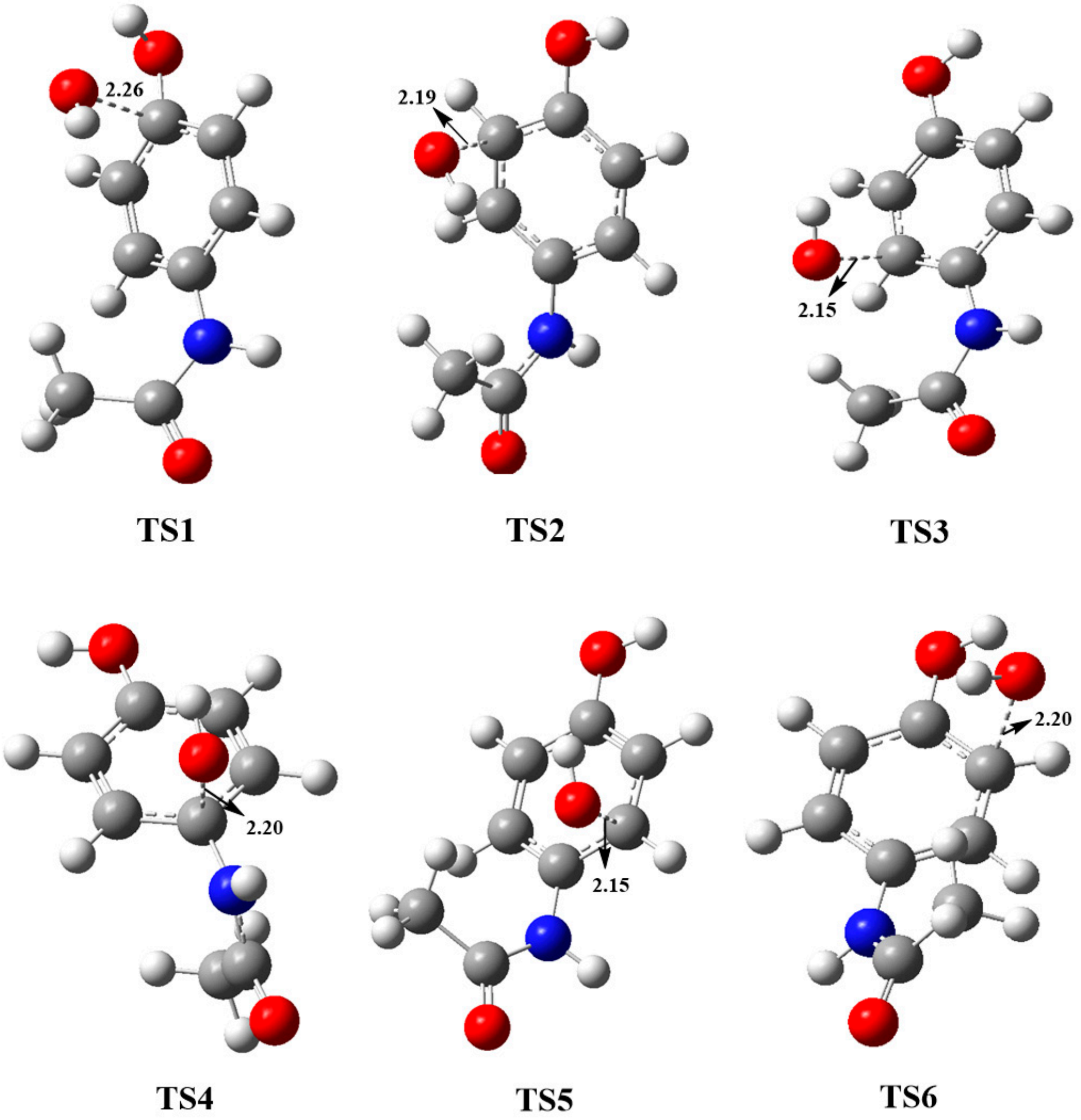

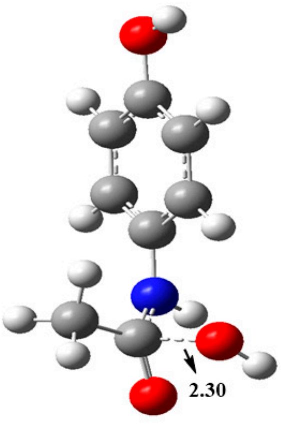

TS7

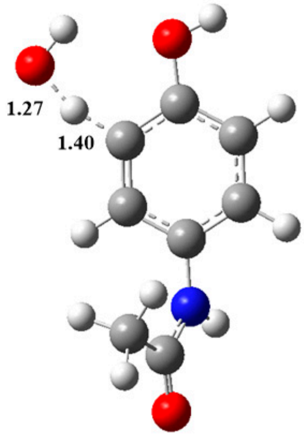

HTS15

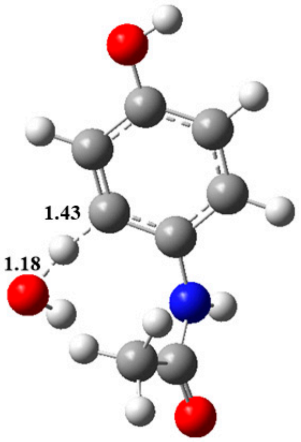

HTS16

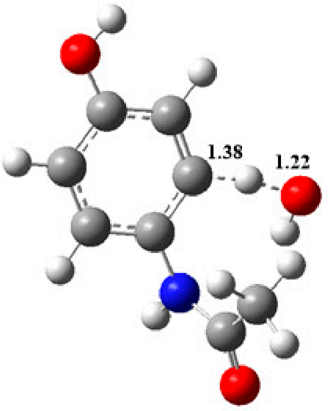

HTS17

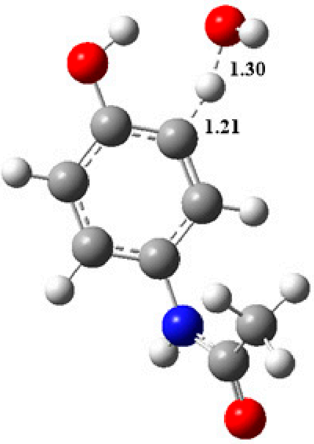

HTS18

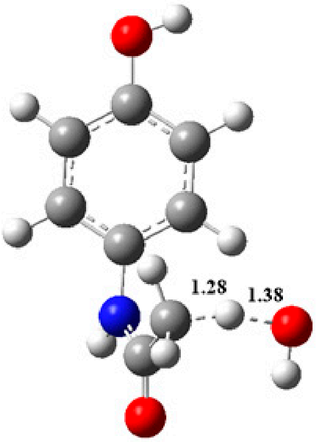

HTS19

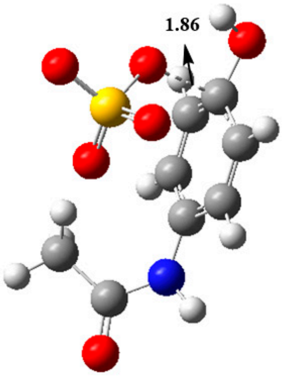

TS8

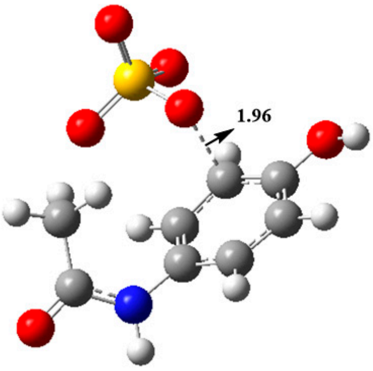

TS9

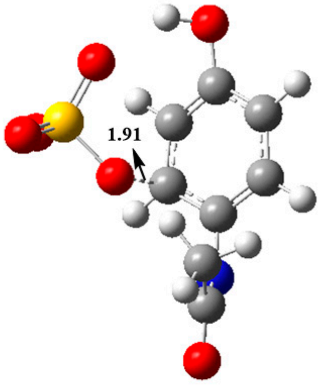

TS10

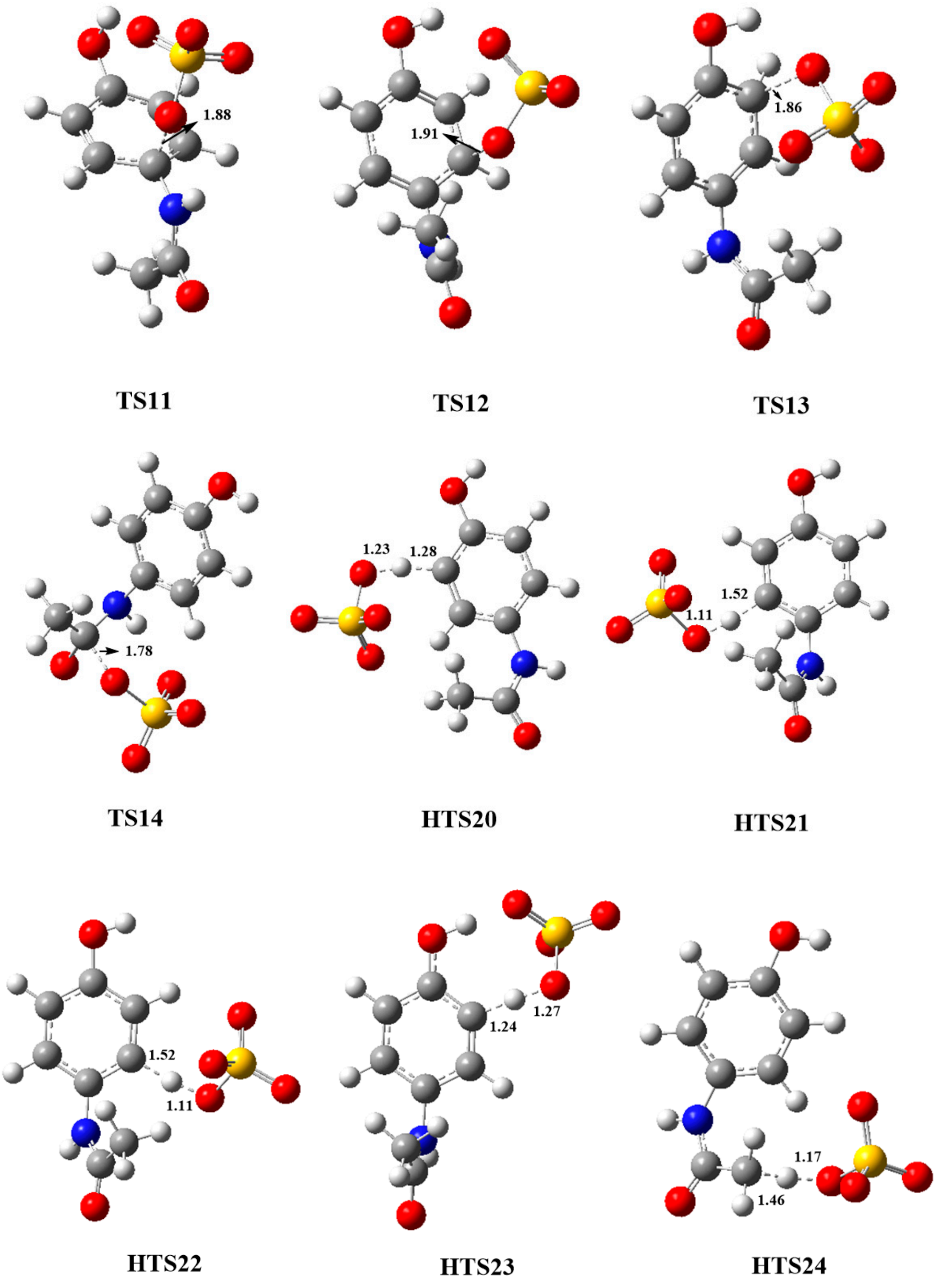

Figure S1. Optimized geometries involving the transition states of AAP with •OH and $SO_4^{\bullet-}$ at the M06-2X/6-31+G(d,p) level. Here,
=H =C =O =S =N.

Table S1. Calculated rate constants of AAP with •OH from 198 to 338 K and 1 atm.

**Paths**	**198**	**218**	**238**	**258**	**278**	**298**	**318**	**338**
Path1	8.04E+07	9.44E+07	8.82E+07	8.42E+07	8.17E+07	8.04E+07	7.99E+07	8.00E+07
Path2	1.87E+08	2.49E+08	2.24E+08	2.07E+08	1.95E+08	1.87E+08	1.82E+08	1.79E+08
Path3	1.51E+07	1.25E+07	1.30E+07	1.36E+07	1.43E+07	1.51E+07	1.59E+07	1.68E+07
Path4	3.22E+08	5.08E+08	4.32E+08	3.81E+08	3.46E+08	3.22E+08	3.04E+08	2.92E+08
Path5	3.33E+07	3.51E+07	3.40E+07	3.34E+07	3.32E+07	3.33E+07	3.36E+07	3.41E+07
Path6	9.02E+09	7.84E+09	6.01E+09	4.86E+09	4.10E+09	3.56E+09	3.18E+09	2.90E+09
Path7	6.75E+02	2.04E+01	6.00E+01	1.51E+02	3.36E+02	6.75E+02	1.25E+03	2.17E+03
Path15	7.36E-04	7.95E-03	5.80E-02	3.14E-01	1.34E+00	4.75E+00	1.44E+01	3.88E+01
Path16	4.40E-03	3.55E-02	2.03E-01	8.95E-01	3.21E+00	9.75E+00	2.60E+01	6.20E+01
Path17	4.51E-04	4.36E-03	2.89E-02	1.44E-01	5.74E-01	1.91E+00	5.51E+00	1.41E+01
Path18	1.28E+02	4.16E+02	1.11E+03	2.57E+03	5.30E+03	1.15E+04	1.75E+04	2.89E+04
Path19	9.61E+01	3.51E+02	1.03E+03	2.59E+03	5.73E+03	1.13E+04	2.11E+04	3.65E+04
$k_{total\text{-}aq}$	9.65E+09	8.74E+09	6.80E+09	5.58E+09	4.77E+09	4.20E+09	3.80E+09	3.51E+09

Table S2. Calculated rate constants of AAP with $SO_4^{\bullet -}$ from 198 to 338 K and 1 atm.

**Paths**	**198**	**218**	**238**	**258**	**278**	**298**	**318**	**338**
Path8	9.43E+16	6.50E+15	7.13E+14	1.12E+14	2.32E+13	6.00E+12	1.86E+12	6.67E+11
Path9	2.08E+14	2.85E+13	5.53E+12	1.40E+12	4.37E+11	1.61E+11	6.78E+10	3.19E+10
Path10	1.76E+14	2.87E+13	6.44E+12	1.85E+12	6.43E+11	2.60E+11	1.19E+11	6.04E+10
Path11	2.14E+13	3.50E+12	7.92E+11	2.29E+11	8.03E+10	3.28E+10	1.51E+10	7.74E+09
Path12	1.70E+14	2.78E+13	6.24E+12	1.79E+12	6.23E+11	2.52E+11	1.16E+11	5.85E+10
Path13	5.65E+18	2.66E+17	2.12E+16	2.53E+15	4.13E+14	8.65E+13	2.22E+13	6.76E+12
Path14	1.46E-11	3.47E-10	4.91E-09	4.67E-08	3.26E-07	1.77E-06	7.86E-06	2.95E-05
Path20	3.98E-02	1.93E-01	7.25E-01	2.25E+00	6.02E+00	1.43E+01	3.07E+01	6.10E+01
Path21	1.18E+00	4.02E+00	1.12E+01	2.69E+01	5.75E+01	1.11E+02	2.00E+02	3.37E+02
Path22	2.81E+00	8.72E+00	2.25E+01	5.06E+01	1.02E+02	1.88E+02	3.23E+02	5.24E+02
Path23	1.70E-02	8.03E-02	2.96E-01	9.03E-01	2.38E+00	5.55E+00	1.18E+01	2.31E+01
Path24	4.59E+09	1.71E+09	7.59E+08	3.84E+08	2.16E+08	1.33E+08	8.70E+07	6.05E+07
$k_{total\text{-}aq}$	5.74E+18	2.73E+17	2.19E+16	2.65E+15	4.38E+14	9.32E+13	2.44E+13	7.59E+12

Table S3. The acute and chronic toxicity class (mg L^{-1}).

**Classification**	**Acute toxicity**[1]	**Chronic toxicity**[2]
**Not harmful**	LC_{50}>100 or EC_{50}>100	ChV>10
**Harmful**	10< LC_{50} <100 or 10< EC_{50} <100	1< ChV <10
**Toxic**	1< LC_{50} <10 or 1< EC_{50} <10	0.1< ChV <1
**Very toxic**	LC_{50}<1 or EC_{50}<1	ChV<0.1

[1]Criteria set by the European Union (described in Annex VI of Directive 67/548/EEC);.
[2]Criteria set by the Chinese hazard evaluation guidelines for new chemical substances (HJ/T 154–2004).

Table S4. Eco-toxicity values of AAP and its transformation intermediates to aquatic organisms (mg L^{-1}).

		**AAP**	**IM1**	**IM13**	**IM8**	**IM6**
Acute Toxicity	Fish (LC_{50})	323	7.08	186	120	21.4
	Daphnia (LC_{50})	63.1	21.4	933	562	77.6
	Green algae (EC_{50})	26.3	3.47	0.73	34.7	64.6
Chronic Toxicity (ChV)	Fish	26.3	5.50	6.2×10^3	2.0×10^3	64.6
	Daphnia	5.13	1.95	602	269	14.8
	Green algae	37.2	5.50	692	347	29.5
